# Bipolar switching in chalcogenide phase change memory

**DOI:** 10.1038/srep29162

**Published:** 2016-07-05

**Authors:** N. Ciocchini, M. Laudato, M. Boniardi, E. Varesi, P. Fantini, A. L. Lacaita, D. Ielmini

**Affiliations:** 1 Dipartimento di Elettronica, Informazione e Bioingegneria, Politecnico di Milano, Piazza L. da Vinci 32–20133 Milano, Italy; 2 Micron Technology, Via Torri Bianche 24, 20871 Vimercate (MB), Italy

## Abstract

Phase change materials based on chalcogenides are key enabling technologies for optical storage, such as rewritable CD and DVD, and recently also electrical nonvolatile memory, named phase change memory (PCM). In a PCM, the amorphous or crystalline phase affects the material band structure, hence the device resistance. Although phase transformation is extremely fast and repeatable, the amorphous phase suffers structural relaxation and crystallization at relatively low temperatures, which may affect the temperature stability of PCM state. To improve the time/temperature stability of the PCM, novel operation modes of the device should be identified. Here, we present bipolar switching operation of PCM, which is interpreted by ion migration in the solid state induced by elevated temperature and electric field similar to the bipolar switching in metal oxides. The temperature stability of the high resistance state is demonstrated and explained based on the local depletion of chemical species from the electrode region.

Nonvolatile memory technology has been historically associated with charge trapping in flash memory, where electrons are stored in a floating gate^1^. As the flash memory scaling approaches the 10 nm node, novel materials and concepts are explored to provide alternative avenues for high density memory technology^2^. Among these alternative concepts, phase change memory (PCM) relies on chalcogenide materials being capable of changing their structural phase by electrical pulses inducing local Joule heating^3^,^4^. Based on the different band structure and resistivity, the amorphous and crystalline phases can be distinguished simply by reading the electrical current^5^. PCM has demonstrated ultrafast (<1 ns) switching by suitable design of the programming pulse^6^ and low-current (<10 μA) operation thanks to carbon nanotube electrode with few-nm dimension^7^,^8^. PCM has reached commercial success and is considered today a mature technology, as demonstrated by proposed applications as a switch in radiofrequency (RF) power transmission^9^, neuromorphic networks^10^, or digital circuits^11^.

One of the key limitations of PCM is the temperature stability of the amorphous phase, which exhibits structural relaxation (SR) and crystallization even at low temperature. SR causes a broadening of the energy gap and a consequent increase of the amorphous-phase resistivity^12^,^13^, while crystallization results in a steep decay of resistance^14^. Both these physical phenomena pose limitation to the use of PCM in high-temperature environments, such as those in many automotive and industrial applications. To enhance the temperature stability of PCM, alternative materials and/or physical concepts should be explored. Non-thermal switching concepts in PCM include interface switching^15^ and electromigration^16^, where electrical pulses affect the structure of defects in the material rather than inducing heating for thermally-activated phase transformation. Field-induced ionic migration finds wide application in oxide-based resistive-switching memory (RRAM), where the migration of ionized oxygen vacancies and metallic impurities causes variation of conductance in either a localized filament^17^ or the bulk^18^. Resistive switching in RRAM is typically induced by *bipolar switching*, where the alternative application of positive and negative voltage pulses causes the formation/dissolution of a conductive (or insulating) region via ionic migration^19^. Ionic migration in both the liquid^20^ and the crystalline phases^21^ was previously observed in phase change materials, thus suggesting that PCM can be operated in the bipolar-switching mode with enhanced temperature stability. Here, we demonstrate bipolar switching in PCM based on solid-phase ionic migration allowing for strongly improved reliability characteristics of the device at elevated temperature.

## Results

### Bipolar switching characteristics of PCM devices

**Figure 1:**
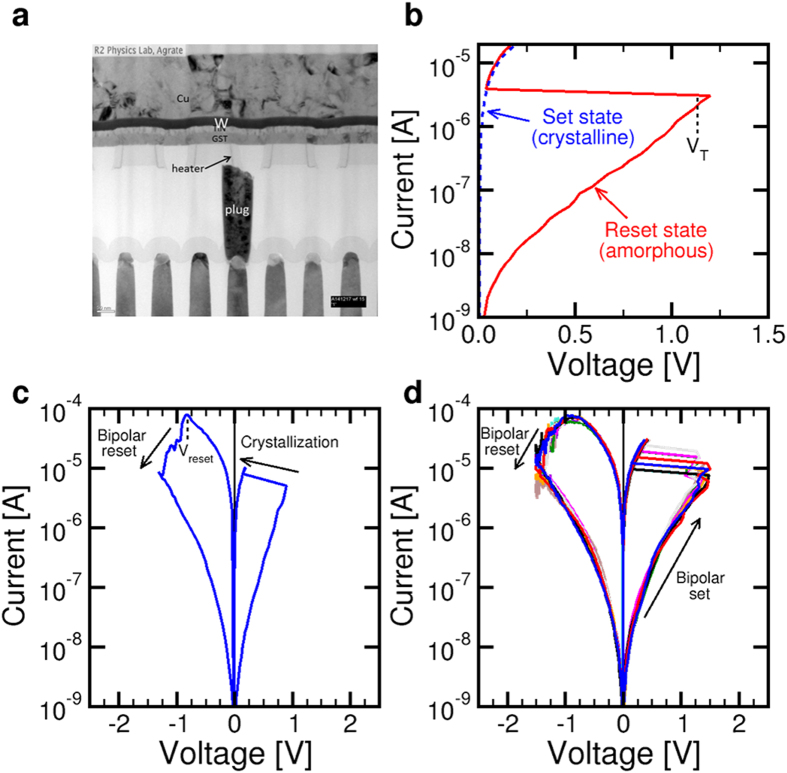
PCM device and characteristics. (**a**) Cross section TEM of a PCM device, indicating the structure with confined BE. (**b**) Measured I-V curve for the 2 states obtained by conventional phase change, namely the set state (crystalline phase) and the PR state (amorphous phase). (**c**) Measured I-V curves for the PCM device under bipolar switching: A first positive voltage sweep is applied to an initially PR (amorphous) state to induce crystallization of GST within the cell, then a negative sweep is applied inducing bipolar reset. (**d**) Measured I-V curves for bipolar switching following the initialization of (**c**) and displaying similar bipolar set and bipolar reset operations of the device for 10 cycles.

Figure 1a shows the experimental PCM device that was used to study bipolar switching. The active material was Ge_2_Sb_2_Te_5_ (GST), which is considered a standard material for optical storage and electrical memory devices. The phase change material was sandwiched between two electrodes, a confined bottom electrode (BE), or heater, and a thin film top W electrode. The confined geometry of the BE allows effective localization of electrical current for efficient Joule heating. PCM operation by phase transformation was demonstrated by electrical pulses (see Fig. S1 in the Supplementary Information). Figure 1b shows the measured current-voltage (I-V) curve for the positive reset (PR) state, which was obtained by application of a positive pulse above melting followed by sudden quench resulting in a disordered amorphous structure of GST. All voltages are referred to the top electrode with the BE grounded. The I-V curve shows high resistance and threshold switching^22^ at a characteristic voltage V_T_. The I-V curve for the set (crystalline) state is also shown, demonstrating a smaller resistance with no threshold switching. Note that positive I-V curves were obtained by applying a current and measuring the voltage across the device, while the negative I-V curves were obtained by applying a voltage and measuring the current through the device. This is a standard technique in bipolar switching, which is needed to limit the current during set transition and the voltage during reset transition, respectively, thus avoiding destructive breakdown of the device.

To test bipolar switching operation, we applied a sequence of positive and negative sweep to the amorphous PR state as shown in Fig. 1c. While the positive sweep causes set transition, namely full crystallization and a consequent decrease of resistance, the negative pulse induces reset transition, evidenced by the increase of resistance in correspondence of a reset voltage V_reset_ = −0.8 V. Note that this reset transition cannot be ascribed to amorphization, since this would require a fast quenching rate in the range of 10–100 K/ns corresponding to about 100 mV/ns, which is approximately 10^9^ faster than our sweep rate in the figure. Another significant difference between bipolar switching and the conventional phase change switching is the resistance of the bipolar reset state (about 1 GΩ in Fig. 1c), which was remarkably higher than the PR state (typically around 30 MΩ for our device, see Fig. 1b).

We verified that the bipolar switching operation is repeatable by re-applying the same positive/negative sweeps for 10 times in Fig. 1d, evidencing similar set/reset processes. The remarkable difference between the first sweep initializing bipolar switching and any subsequent sweeps is that the values of V_T_ and V_reset_ are larger while the resistance in the set state is smaller in the first sweep. These differences can be attributed to the set and reset states in bipolar switching being different from the amorphous and crystalline phase in the usual phase change switching. We verified that bipolar switching can be initiated from a PR state (amorphous phase) already by a negative sweep, i.e., without passing to a crystalline set state (see Supplementary Fig. S2). The bipolar switching curves observed for different initial state (either crystalline or amorphous states) show similar behavior in terms of resistance values and switching voltages. In all cases, the bipolar switching characteristics show a strong similarity with typical curves for metal-oxide RRAM devices^22^.

### Nanoscale redistribution of atomic species

**Figure 2:**
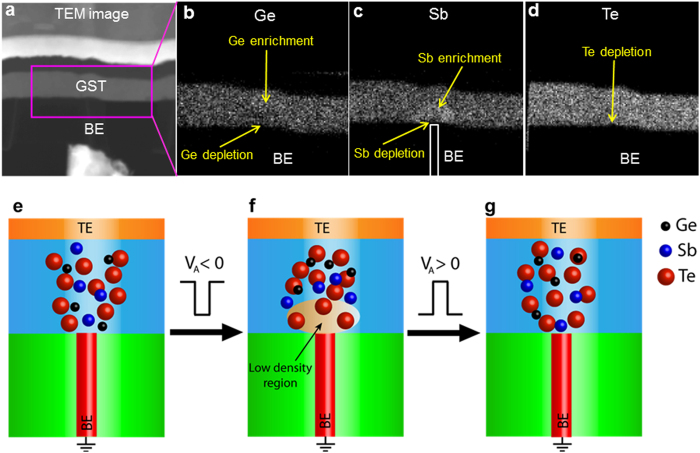
Microscopic switching mechanism. (**a**) Cross section TEM of the PCM device highlighting the region investigated for elemental analysis after bipolar reset. (**b**,**c**,**d**) EDX profiles of Ge, Sb and Te, respectively, showing local depletion of Ge and Sb in correspondence of the BE. (**e,f,g**) Pictorial view of the elemental profile in the initial set state (**e**), after bipolar reset inducing ionic migration and species separation causing local depletion at the BE interface (**f**) and after bipolar set responsible for redistribution of the chemical elements within the GST volume.

To understand the origin of bipolar switching in PCM and of the high resistance after bipolar reset, we carried out elemental analysis in the active layer in a PCM after bipolar reset. Figure 2 shows the cross-section transmission electron micrograph (TEM) for the PCM device (a) and the energy dispersive x-ray spectroscopy (EDX) analysis for Ge (b), Sb (c) and Te (d). Ge shows a local depletion close to the BE interface and an enrichment in the upper half of the GST film, which is indicative of Ge moving away from the BE during bipolar reset. This agrees with the cation behavior of Ge, which migrates toward the negatively-biased top electrode during bipolar reset. Sb shows a similar depletion close to the BE, while the Sb enrichment is still confined in the lower half of the GST film, hence closer to the BE. Finally, Te does not show any obvious non-uniform profile within the active region. Note that the element profile is different from the one observed after set transition under applied positive voltage^23^, showing extensive accumulation of Sb close to the BE. This suggests that, due to the large electric field and the high local temperature assisting ionic migration, electropositive species, such as Ge and Sb, are attracted away from the positively-biased BE during bipolar reset. Therefore we attribute the bipolar reset process to the transition from almost uniformly distributed elements in the amorphous/crystalline GST operated by conventional phase change (Fig. 2e), to a strong depletion in the BE region, particularly of Sb and Ge, which leads to a high local concentration of defects, such as vacancies, possibly leading to a vacuum tunnel barrier (Fig. 2f). Application of a positive sweep induces redistribution of Sb and Ge back to the depletion layer, thus contributing to a uniform elemental profile and a lower resistance (Fig. 2g). This is similar to the previously observed electromigration-driven dislocation jamming in GST nanowire, although we attribute the high value of resistance in the bipolar reset state to a tunneling barrier across the depleted gap, instead of an amorphous layer. Note that, although Te is expected to behave as an anion migrating toward the positively biased BE during bipolar reset, the Te profile shows an almost uniform distribution in Fig. 2d. This result is in agreement with Te profiles measured after set operation under positive voltage in PCM devices operated with conventional unipolar phase change operation^23^. Such a low participation of Te in the migration process might be due to a low average ionization charge, or to a low ionic mobility of Te.

### Pulsed bipolar switching characteristics

**Figure 3:**
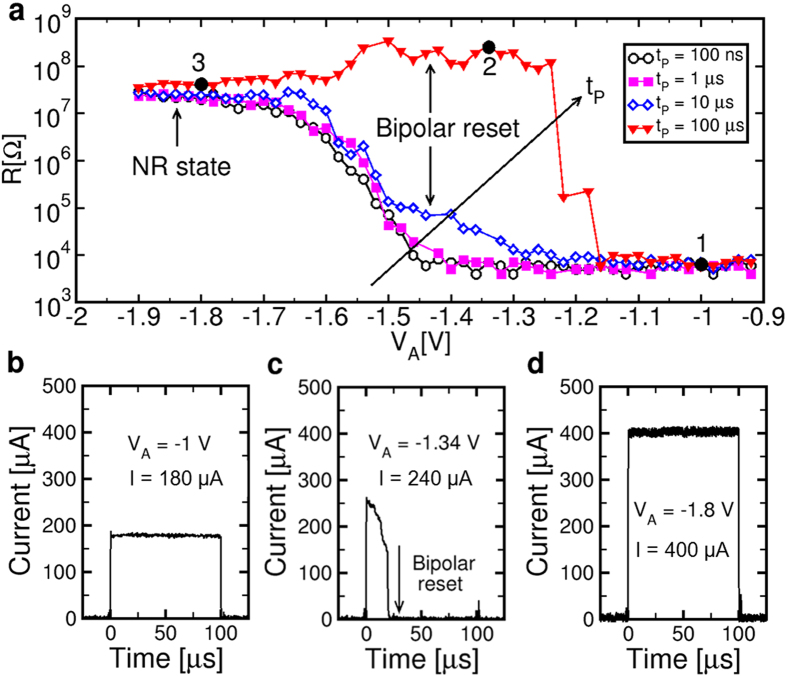
Pulsed bipolar reset characteristics. (**a**) R measured after the application of a negative voltage pulse of amplitude V_A_ on a PCM device initially prepared in the set state. Pulses with variable pulse-width t_P_ were applied, from 100 ns to 100 μs. For t_P_ below 10 μs, resistance increases above the melting point due to amorphization. For t_P_ = 10 μs and 100 μs, an additional increase of resistance is observed just below melting, at −1.3 V and −1.2 V, respectively. This additional feature can be attributed to ionic migration and the formation of a depleted barrier with high resistance close to the BE interface. (**b**–**d**) Waveforms of the PCM current during pulses indicated as points 1, 2 and 3, respectively, in (**a**). The current remains low for V_A_ = −1 V (**b**), shows a sudden drop indicative of bipolar switching for V_A_ = −1.34 V (**c**) and remains equal to a high value for V_A_ = −1.7 V.

Bipolar switching can be operated in the pulsed mode, similar to oxide-based RRAM^24^. Figure 3a shows the bipolar reset characteristic under pulsed conditions, namely the resistance R measured after the application of a negative pulse, as a function of the applied pulse voltage V_A_. The device was always initialized into a crystalline set state with resistance around 30 kΩ by application of a PR pulse for amorphization (see Fig. S1) followed by a positive sweep for crystallization. Increasing pulse-widths were used from t_P_ = 100 ns to 100 μs. Data show that, for relatively short pulse-widths below 10 μs, amorphization takes place around −1.4 V, where R gradually increases from the crystalline value to the amorphous value where it saturates around −1.7 V. This is due to the thermally-induced melting and quenching, resulting in a volume of GST being transformed into the amorphous phase. The amorphous high-R state obtained by a short negative voltage pulse will be referred to as the negative reset (NR) state, since it shares the same structure as the PR state, namely an amorphous phase obtained by quenching from the liquid phase. The NR characteristic is further compared to the PR characteristic in Fig. S1, where the higher voltage needed to induce melting under negative voltage is due to the thermoelectric effects in PCM^25^. A similar characteristic is obtained for t_P_ = 10 μs, except for the bump-like feature where R increases from a voltage just below the melting point, namely around −1.3 V. The bump becomes even more evident at t_P_ = 100 μs, where R rises to the large value of around 0.3 GΩ which is typical of the bipolar reset state. The bump region at t_P_ = 100 μs extends approximately between −1.2 V and −1.6 V, where the resistance decreases to the NR state. We attribute the bump around −1.3 V to bipolar reset process, namely the same ion-migration process taking place in the dc I-V curves of Fig. 1c,d under negative voltage and summarized in Fig. 2f. Note that the bump due to bipolar reset vanishes at large negative voltages well within the melting regime, where resistance decreases to the typical NR value. This suggests that bipolar reset can only take place in the solid state, because the depleted gap would not be sustained above melting due to the insufficient viscosity of the liquid phase. Also, the bipolar reset pulse arises only for relatively long t_P_ , since shorter t_P_ would not be enough to complete the ion migration process in GST and the formation of a sufficient defect concentration in the depletion layer.

The pulsed-mode bipolar reset was studied by monitoring the current during the applied voltage pulses for t_P_ = 100 μs: at low voltage (point 1 at V_A_ = −1 V), the current remains a relatively low values below 200 μA with no transition due to either phase change or ion migration (Fig. 3b). At moderate high voltage (point 2 at V_A_ = −1.34 V), the current exhibits a sharp drop at t = 20 μs, which we identify with the bipolar reset transition where the depleted gap with high R is formed (Fig. 3c). Finally, at high voltage above melting (point 3 at V_A_ = −1.8 V), the current remains at relatively high values above 400 μA with no discontinuities (Fig. 3d), which can be attributed to the metallic conductivity of the liquid GST above the melting point^26^. As the voltage is suddenly turned off at the end of the pulse, the liquid phase transforms into the amorphous phase with high R in the NR state. These results further confirms that bipolar reset is only possible in the solid state, since no sharp drop of resistance is observed above melting. At the transition point between bipolar reset and NR (around V_A_ = −1.55 V in Fig. 3a), we observed random current oscillations instead of the simple drop of resistance (see Supplementary Fig. S3), which can be explained by a temperature/structure instability where the opening of a depleted gap results in a higher temperature causing melting and collapse of the depleted gap. These results support our interpretation of bipolar reset as due to ion migration in the solid phase.

A behavior similar to Fig. 3b–d was seen in response to a positive voltage pulse of 100 μs duration applied to a bipolar reset state (see Supplementary Fig. S4). For relatively low voltage V_A_ = 1 V, the current remains low in the off state (Fig. S4a). As the voltage is increased to 1.1 V (Fig. S4b), the current shows an abrupt increase after about 80 μs from the start of the pulse, indicating the bipolar set transition in the PCM. Finally, an applied voltage of 1.25 V (Fig. S4c) causes switching at shorter time during the pulse. Note that also the on-state current after set transition increases, probably due to the higher voltage and to the larger decrease of resistance at high voltage as a consequence of the stronger ionic migration. We also verified that the device can operate repeatedly under bipolar switching with positive set and negative reset (see Supplementary Fig. S5). The device showed up to 10^4^ cycles with no signs of degradation, where each cycle included a triangular set pulse with maximum voltage V_A_ = 1.55 V and pulse width t_P_ = 100 μs, followed by a triangular reset pulse with maximum negative voltage V_A_ = −1.4 V and t_P_ = 200 μs. These results support the common nature of bipolar set and reset processes by ion migration and demonstrate the feasibility of bipolar switching for PCM operation.

### Temperature dependence of bipolar reset

**Figure 4:**
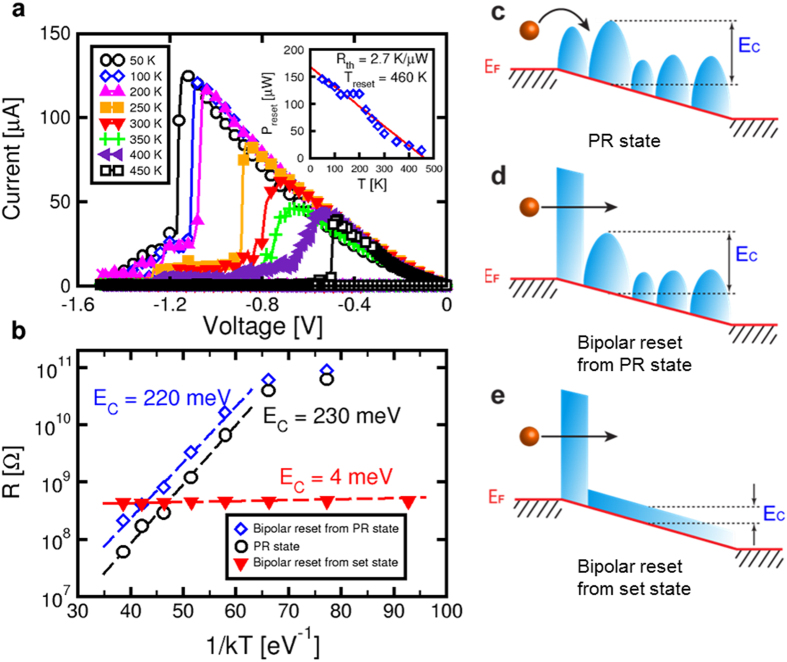
Temperature dependence of switching and transport. (**a**) Measured I-V curves for bipolar reset at variable temperatures, showing that both the reset voltage and current decrease at increasing T. The inset shows the measured reset power as a function of ambient temperature T_0_: the linear fitting allows to extract the thermal resistance R_th_ = 2.7 KμW^−1^ and the reset temperature T_reset_ = 460 K. (**b**) Arrhenius plot of resistance for the bipolar reset states obtained either starting from the PR state (amorphous phase) or from the set state (crystalline phase). The Arrhenius plot of the PR state is also shown for reference. The activation energy E_C_ of the bipolar reset state depends on the previous state because regions of amorphous or crystalline phase remain in GST after bipolar reset. (**c**,**d**,**e**) Band diagrams for the PR state, the bipolar reset state obtained from a PR state, and the bipolar reset state obtained from the set state, respectively. Transport in the PR state is controlled by PF emission over an energy barrier E_C_, while the tunneling barrier controls R for bipolar reset states. However, depending on the residual amorphous (**d**) or crystalline phase (**e**) in GST, different values of E_C_ are obtained.

Figure 4a shows measured I-V curves during bipolar reset at increasing ambient temperature T_0_ = 50 K to 450 K. As T_0_ increases, the reset voltage V_reset_ decreases, which can be understood by the temperature-activation of ionic migration^24^,^27^. To activate ionic migration within the experimental time, in fact, a certain local temperature T must be reached in the GST volume by Joule heating. As T_0_ increases, less voltage and current are needed to reach the critical T_reset_ needed for effective ion migration. The inset of Fig. 4a shows the reset power P_reset_, obtained by multiplication of the voltage and current at the reset point in the characteristics of Fig. 4a, as a function of the ambient temperature T_0_. Data show a linearly decreasing P_reset_ consistent with the analytical formula^17^,^28^,^29^:


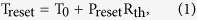


where R_th_ is the effective thermal resistance describing the ratio between the local temperature increase in GST and the dissipated power. From the linear fitting of experimental P_reset_ and T_0_, we obtain R_th_ = 2.7 KμW^−1^ and T_reset_ = 460 K, in good agreement with previous results for the same PCM technology^28^. Results for the thermal resistance in other experimental samples are markedly smaller, namely R_th_ = 1.6 KμW^−1^ obtained for PCM melting^29^. The larger R_th_ in our device can be understood by the stronger heat confinement due to the ultrathin BE of about 5 nm (see Fig. 1a). The relatively low T_reset_ is due to the low sweep rate (around 1 Vs^−1^) in the I-V curves of Fig. 4a: Due to the relatively long time of the measurement, a low temperature was needed to initiate ion migration at the origin of bipolar switching. Note that T_reset_ in our PCM is comparable to the value of about 530 K observed in NiO RRAM^30^, further supporting the similarity with migration-induced switching in RRAM.

### Temperature dependence of resistance

We studied the temperature dependence of the resistance to confirm our picture of the bipolar reset state in Fig. 2f. To this purpose, we prepared a device in the bipolar reset state by application of a negative sweep as in Fig. 1c, either starting from a set state (crystalline phase) or a PR state (amorphous phase). Figure 4b shows the Arrhenius plot of R for bipolar reset state obtained from crystalline or amorphous phases. Bipolar reset states show different activation energies E_C_ of resistance, namely E_C_ = 0.22 eV for bipolar reset applied to the amorphous phase and E_C_ = 4 meV for bipolar reset applied to the crystalline phase. The value of E_C_ for the bipolar reset state starting from an amorphous phase is similar to that of the PR state (E_C_ = 0.23 eV), which is reported for reference in Fig. 4b. The characterization of the temperature dependence of conduction for many PCM devices gave results consistent with Fig. 4b (see Supplementary Fig. S6). The different E_C_ in Fig. 4b can be explained by the band diagrams in Fig. 4c–e: the PR state is described by localized potential wells associated with disorder and defects in the amorphous phase (Fig. 4c)^5^. Electrons move from one well to the other by thermally-activated Poole-Frenkel (PF) emission with activation energy given by the energy barrier between the conduction band edge and the Fermi level E_F_ (ref. 5). If bipolar reset is applied to the amorphous phase, a depletion layer is formed at the BE side, where conduction is limited by a tunneling barrier (Fig. 4d). Since tunneling has negligible temperature activation, PF emission still controls the activation energy E_C_, which is thus similar to the PR state. On the other hand, if bipolar reset is operated on the crystalline phase (Fig. 4e), the energy barrier is the one of the crystalline phase, which is known to be in the range of few meV. These results suggest that the depletion layer might act as a tunneling gap at the BE interface.

**Figure 5:**
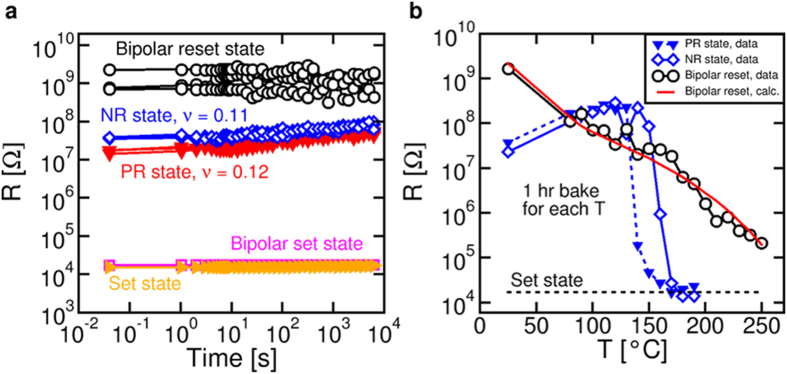
Time and temperature stability. (**a**) Measured R at room temperature as a function of time for bipolar set and reset states, and for NR, PR and set states. Set state and bipolar set state show stable resistance, while NR and PR states show drift as expected from SR in amorphous GST. The bipolar reset state is instead stable over time, confirming that the high R in the bipolar reset state is due to a tunneling barrier rather than an amorphous phase. (**b**) Measured R after an annealing bake as a function of the annealing temperature for PR, NR and bipolar reset states. R was measured at room temperature after each annealing step. NR and PR states display drift and decay due to crystallization around 150 °C, while the bipolar reset state shows a gradual decrease of R with no evidence for drift or crystallization. Calculation results based on the distributed energy model are also shown.

From all previous results, it is clear that the resistance states obtained by bipolar switching are different from the conventional crystalline and amorphous states of the PCM. In particular, the high resistance is dictated by the tunneling barrier in Fig. 4d,e, rather than the amorphous phase resistivity. Therefore, the time and temperature stability of the high resistance state, which is a matter of concern for PCM, can be also expected to be different. Figure 5a shows the measured R as a function of time for 5 different states, namely set and reset state obtained from bipolar switching, and set and reset (both PR and NR) states obtained from conventional phase transformation. The time in the figure is measured with respect to the last set or reset operation. Both set states display stable resistance, while R drifts with time for both PR and NR states, according to the power law formula:


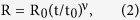


where R_0_ and t_0_ are constants and ν is the drift exponent^12^. Drift in the amorphous phase can be explained by the SR phenomena in the disorder phase, and the observed drift exponent (ν ≈ 0.1) is consistent with other values in the literature for GST^12^. Contrary to NR and PR states, bipolar reset state shows a strikingly stable resistance with no drift, which confirms that the high resistance is not controlled by an amorphous phase resistivity, rather by a tunneling gap which is immune from SR. To further support the enhanced stability of the bipolar reset state, Fig. 5b shows the R measured after annealing at increasing temperature from 20 °C to 250 °C for PR, NR and bipolar reset states. The device was kept at elevated temperature for one hour, then the resistance was measured at room temperature at the end of each annealing step. Both NR and PR states show drift at relatively low temperature below 120 °C, followed by a marked drop due to crystallization of the amorphous phase^13^. On the other hand, R decreases gradually with no evidence for drift or crystallization for the bipolar reset state. It is notable that, even for the highest temperature of 250 °C explored in Fig. 5b, R does not decrease below a factor 10 higher than the set state, thus confirming a high temperature stability of GST under bipolar switching operation. The higher stability can be attributed to resistance being controlled by the tunneling barrier, which is immune from crystallization effects. The decrease of the resistance might be due to back-diffusion of Sb and Ge toward the BE region, which decreases the local concentration of defects and the thickness of the depleted barrier. The annihilation process has been described by a distributed energy barrier model (see Supplementary Fig. S7), for which calculation results are shown in Fig. 5b.

## Discussion

Our results allow to generalize the concept of bipolar switching, which was previously associated to migration of ionized species in 2 well-defined materials systems: (i) metal oxides, where the resistance change is mainly attributed to migration of oxygen vacancies, and (ii) electrochemical metallization systems, where the resistance change is due to the migration of metal cations supplied by an oxidizable electrode^31^. In both these RRAM systems, bipolar switching is localized at a confined conductive filament, which is repeatedly connected and disconnected by the alternation of positive and negative voltage thanks to local ionic motion. In our PCM, instead, the resistance change is due to uniform migration of Ge, Sb and Te activated by the applied voltage. We can rule out filamentary localization of resistance change in our PCM, since bipolar reset in Fig. 1c was observed starting from the crystalline state which would provide a low-resistance current path shunting any high-resistance state filament. The high resistance state is instead due to the formation of a low-density layer with high defect concentration acting as a tunnel layer, as depicted in Fig. 2f.

The key enabling mechanism for bipolar switching in our PCM thus appears (i) Joule heating, enabling a high local enhancement of ionic diffusivity, and (ii) the large electric field, acting as a driving force for the ionic migration and the consequent formation/collapse of the depleted layer at the bottom electrode. From this viewpoint, the mushroom structure of the PCM is most favorable for bipolar switching, in that the bottom electrode area where the depleted region is formed during reset is minimized. Also, the bottom electrode interface is where the largest Joule heating takes place in the whole PCM, thanks to its ideal thermal and current confinements^25^. Further design optimization of bipolar-switching PCM is however possible by carefully tuning heating, conduction and migration by a suitable choice of cell geometry, materials and interfaces. For instance, optimization of the PCM switching dynamics may be achieved by the enhancement of the electric field, which is the major driving force for ionic migration in the switching operation of the device. In particular, reducing the thickness of the active GST layer would allow for an increase of the electric field, thus allowing for further reducing the switching times to the 10–100 ns timescale, which is comparable to the operation dynamics of thermal phase change. The GST thickness in our work was 35 nm, which results in F ≈ 0.4 MVcm^−1^ for an applied voltage of 1.34 V (point 2 in Fig. 3a). Assuming that the migration velocity is proportional to F, the GST thickness reduction might result in a significant acceleration of the formation/dissolution of the depleted region. At the same time, thickness reduction might also reduce the migration distance, thus also contributing to a reduction of the switching time. As a reference, the minimum thickness of the oxide layer in metal-oxide RRAM devices is around 5 nm, which ensures fast migration at relatively low voltage. Note that, while thickness reduction allows to increase F, Joule heating is negligibly affected. Excessive Joule heating, in fact, would lead to melting at relatively low voltage, which would prevent the formation of the depleted region (see Fig. S3). Other solutions to optimize the bipolar switching time in PCM include the material engineering, where the chalcogenide composition is engineered to maximize the ionic mobility of the constituent elements in the solid phase. The material and structure optimization for enhanced migration in phase change materials is currently an unexplored topic, which requires dedicated research efforts of atomistic simulations and extensive characterization of materials and devices under elevated electric field.

Recently, electromigration was shown to induce resistance transition by the electric wind of carriers transferring their momenta to pre-existing defects, such as dislocations, in phase change nanowires made of GST (ref. 16) or GeTe^32^. The role of electromigration in bipolar switching is questionable for two reasons: First, the strong similarities of PCM bipolar switching with RRAM bipolar switching in terms of the shape of the I-V curve and the temperature dependence of the reset voltage. Second, the hole wind is expected to cause defect jamming at the BE for positive voltage applied to the top electrode. In fact, dislocations have been evidenced to migrate toward the negative node by *in-situ* experiments on nanowires^16^,^34^. However, the increase of resistance at bipolar reset suggests defect jamming at the positively-biased BE, contrary to the electromigration interpretation. Therefore, the polarity dependence of resistance switching in our results suggests that ionic migration, instead of electromigration, plays the major role at the origin of the bipolar switching in PCM.

The enhanced time and temperature stability of bipolar switching operated PCM in Fig. 5 appears extremely promising to improve device reliability in several high temperature applications where PCM is today not applicable due to GST crystallization and drift. For instance, embedded memories for system-on-chip (SOC) require high temperature stability due to the soldering and packaging processes, where the chip temperature can rise to above 260 °C for few minutes^33^. Also, memory in automotive applications may work at elevated temperature for long time. Bipolar switching can thus be used to provide higher resistance window and higher stability in harsh environments where conventional PCM and RRAM reliability might be challenged.

In summary, we demonstrated bipolar switching in PCM, where the device is operated by application of alternated positive and negative pulses or sweeps. A high resistance approaching 1 GΩ is observed in the bipolar reset state which is attributed to elemental depletion and tunneling barrier in the vicinity of the BE. The strong stability of the device, which is demonstrated by high-temperature annealing studies, paves the way for GST-based PCM devices with enhanced stability at high temperature.

## Methods

The phase change device (PCM) used in this study consists of a two-terminal resistor-type device with a phase change layer enclosed between two metallic contacts, serving as the top and bottom electrodes. The phase change layer was made of a doped Ge_2_Sb_2_Te_5_ (GST) film deposited by RF sputtering^13^. The phase change film was in the amorphous state soon after deposition, then recrystallized completely while completing the electrode deposition and passivation steps at elevated temperature. The top and bottom electrodes were obtained by sputtering of TiN, with a Ti adhesion layer deposited between the phase change material and the top electrode to avoid delamination of the top electrode and to ensure low contact resistance. The BE was laterally defined by conventional lithography to a 50 nm size in one direction and by sub-lithographic technique to 5 nm size in the orthogonal direction. The thickness of the phase change layer was 35 nm. Insulation among adjacent devices was ensured by SiN surrounding each PCM^34^. The electrical characterization was allowed by square contact pads with 20 μm size which were connected to the top and bottom electrodes via metal lines and vias.

The dc conduction and bipolar reset characteristic of PCM were collected by an HP4155B Semiconductor Parameter Analyzer connected to the experimental device within a conventional probe station for electrical characterization. The standard programming characteristics and the pulsed bipolar reset characteristics demonstrations were conducted by using a TTi – TGA 12102 arbitrary waveform generator with a 100 MHz bandwidth to deliver rectangular pulses of different duration. The voltage response of the PCM was captured by an active probe connected to a Lecroy Waverunner oscilloscope with 600 MHz bandwidth and maximum 4 GSample/s sampling rate. A complete electrical characterization of PCM devices is reported in the Supplementary Information.

## Additional Information

**How to cite this article**: Ciocchini, N. *et al*. Bipolar switching in chalcogenide phase change memory. *Sci. Rep.* **6**, 29162; doi: 10.1038/srep29162 (2016).

## Supplementary Material

Supplementary Information
