# Supplementary material for: Bipolar switching in chalcogenide phase change memory
Source: Sci Rep. 2016 Jul 5;6:29162. doi: 10.1038/srep29162 (PMC4932516; doi:10.1038/srep29162)
Supplement: Supplementary Information [file srep29162-s1.pdf]

Supplementary Information

Bipolar switching in chalcogenide phase change memory

N. Ciocchini[1], M. Laudato[1], M. Boniardi[2], E. Varesi[2], P. Fantini[2], A. L. Lacaita[1] & D. Ielmini[1]*

[1]*Dipartimento di Elettronica, Informazione e Bioingegneria, Politecnico di Milano, Piazza L. da Vinci 32 – 20133 Milano, Italy*

[2] *Micron Technology, Via Torri Bianche 24, 20871 Vimercate (MB), Italy*

*Contact: daniele.ielmini@polimi.it

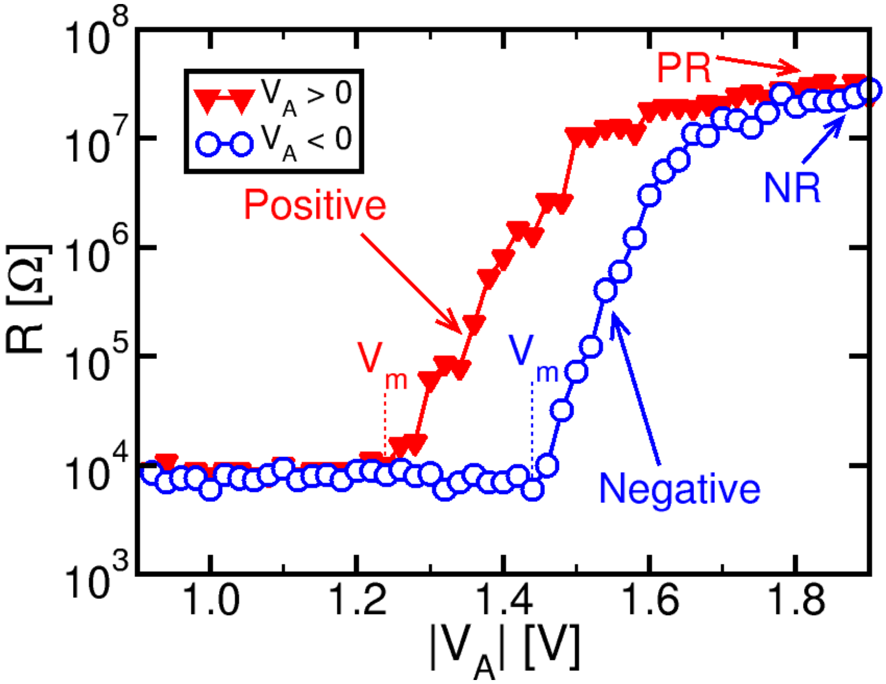

Figure S1 | Programming phase-change characteristics of the PCM. Resistance R of the PCM measured after the application of a pulse as a function of the pulse voltage amplitude V_A. The programming pulse with either positive or negative voltage V_A was applied on a device initially prepared in a set state by application of a positive voltage pulse for crystallization. As $|V_A|$ increases, the resistance increases above the melting voltage for which the chalcogenide layer starts being transformed into the liquid phase and then, after turning off the pulse, into the amorphous phase [1]. At large $|V_A|$, R saturates to the value of the positive reset (PR) state or the negative reset (NR) state, for positive or negative V_A, respectively. The melting voltage is higher for negative pulse compared to the positive pulse, as a results of thermoelectric heating contribution making Joule heating less effective under a negative voltage [2].

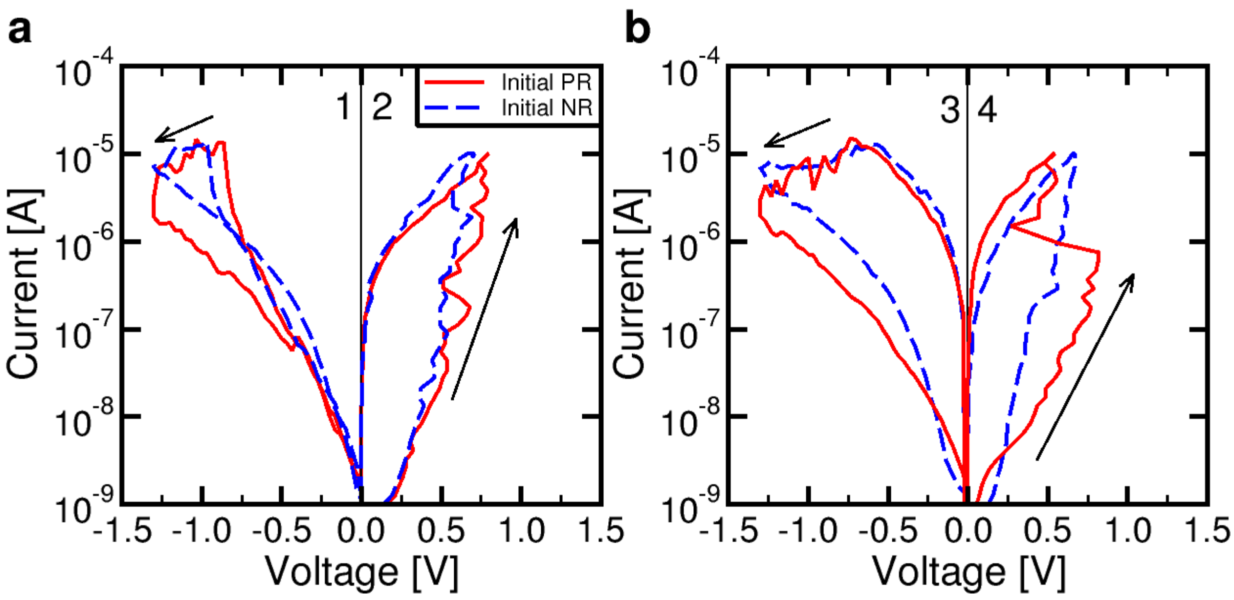

Figure S2 | Bipolar switching characteristics. (a) Measured I-V curves where a first negative voltage sweep is applied on either PR and NR state (1), followed by a second positive voltage sweep (2) for set transition. (b) Measured I-V curves for repeated negative (3) and positive voltages (4) indicating stable bipolar switching. NR and PR states show comparable sweeps during bipolar switching. During the first negative sweep, the current first increases up to about 10 μA, then decreases due to bipolar reset. After the first sweep, the bipolar switching characteristics show the same behavior as for the case where a positive sweep is applied first (see Fig. 2c and d).

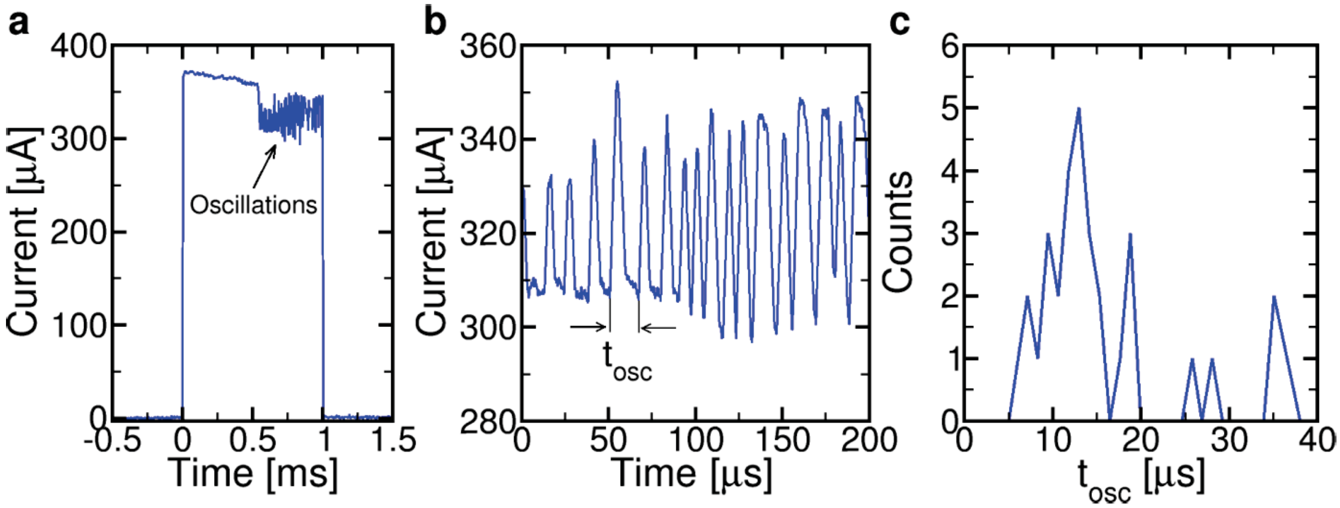

Figure S3 | Current oscillations during bipolar reset at the melting point. (a) Measured current in response to an applied rectangular pulse with amplitude V_A = -1.5 V and pulse-width t_P = 1 ms. (b) Close-up of the current waveform indicating quasi-periodic oscillations with random period t_{osc}. (c) Corresponding distribution of t_{osc}. Current oscillations are explained in terms of alternation of depletion (where the current decreases) and collapse of the tunneling gap in the liquid phase.

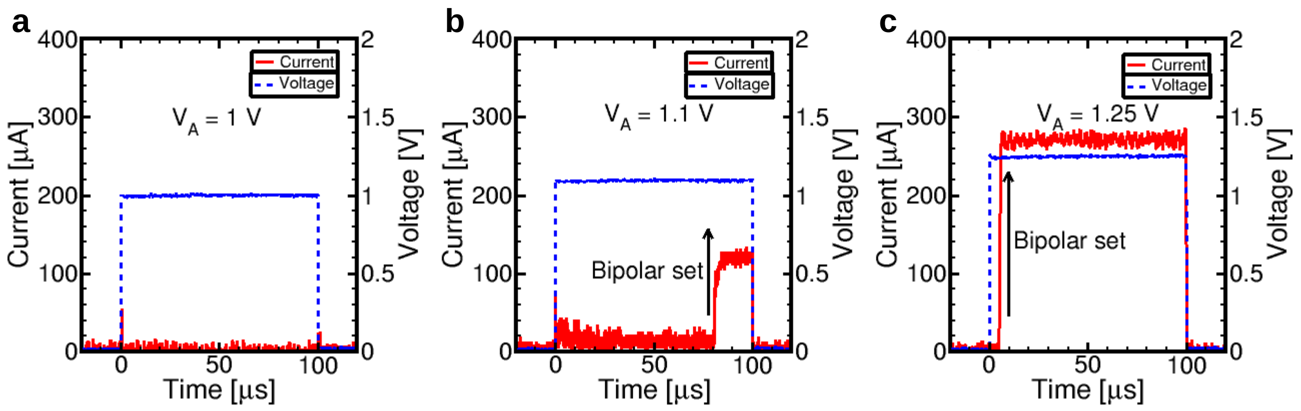

Figure S4 | Current response during bipolar set. Measured current in response to applied rectangular pulses with amplitude V_A = 1 V (a), 1.1 V (b) and 1.25 V (c), all having pulse-width t_P = 100 µs. For relatively low voltage, the current remains in the off-state with no switching (a). At sufficiently large voltage, the current shows an abrupt increase after about 80 µs from the start of the pulse, marking the bipolar set transition in the PCM (b). As the voltage is increased even further, the switching is anticipated to shorter time, while the on-state current increases due to stronger ionic migration and correspondingly larger decrease of resistance (c).

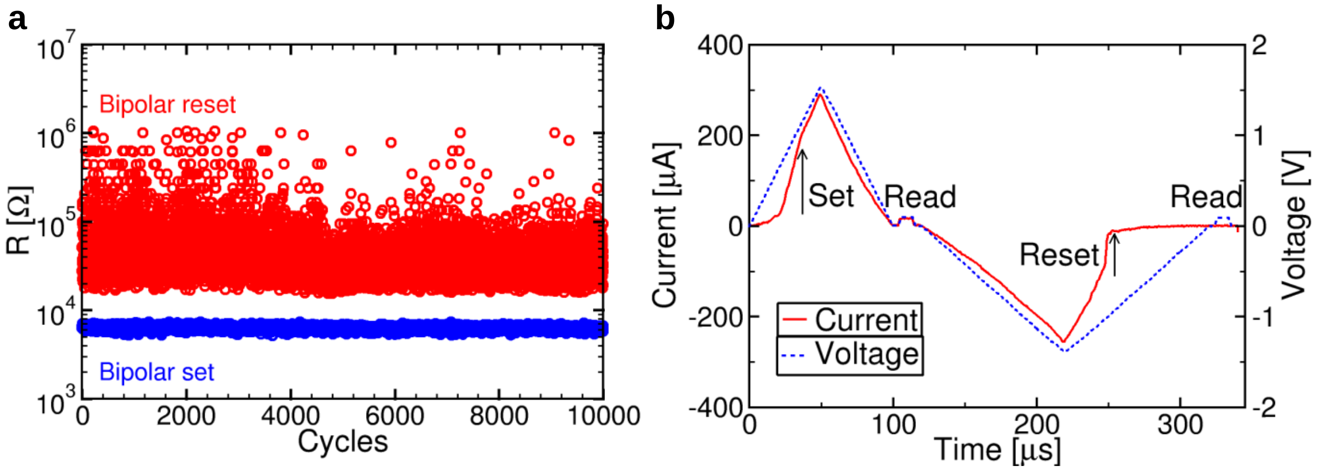

Figure S5 | Bipolar cycling of the device. Measured R after bipolar set and bipolar reset in the pulsed regime for 10^4 cycles (a) and measured applied voltage and current response during a single set/reset cycle (b). We applied triangular pulses of V_A = 1.55 V (pulse width = 100 μs) for bipolar set and V_A = -1.4 V (pulse width = 200 μs) for bipolar reset. The resistance R in the 2 states was measured by a read pulse with pulse-width 10 μs and voltage V_A = 0.1 V, thus low enough to prevent any unwanted disturb to the device. Note that the R window is largely underestimated due to limited current sensitivity in our pulsed setup, where the oscilloscope had a sensitivity of about 20 μV, corresponding to 0.4 μA read current, which limited the capability to evaluate the high R in the bipolar reset state. The inset shows the applied voltage waveform and the corresponding current response during a complete cycle of bipolar set and reset. Note the abrupt drop of current during bipolar reset operation driven by the migration within the active material.

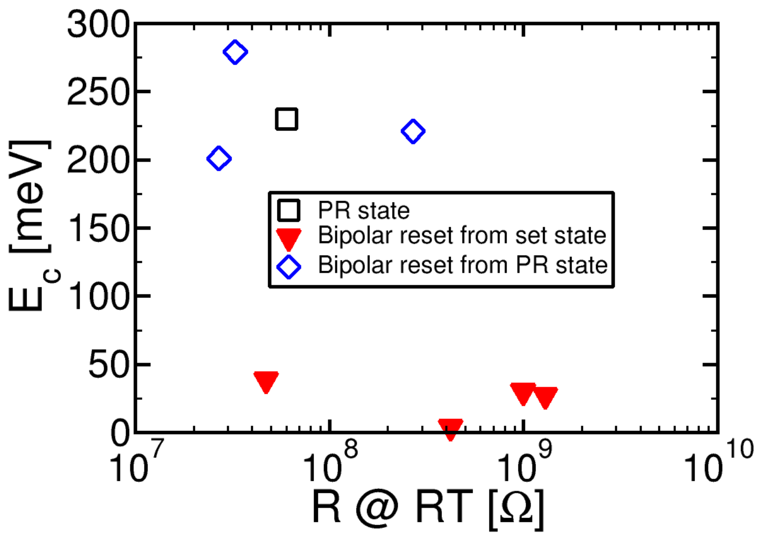

Figure S6 | Activation energy for conduction in PR and bipolar reset state. The activation energy E_C is generally higher than 0.2 eV for PR state (amorphous phase) and bipolar reset state obtained directly applying a negative voltage sweep to a PR state. This is consistent with the resistance R being controlled by the Poole-Frenkel conduction in the amorphous phase. On the other hand, a very small activation energy $E_C < 50$ meV is found for the bipolar reset state obtained from the set state, consistent with the low E_C of conduction in crystalline GST.

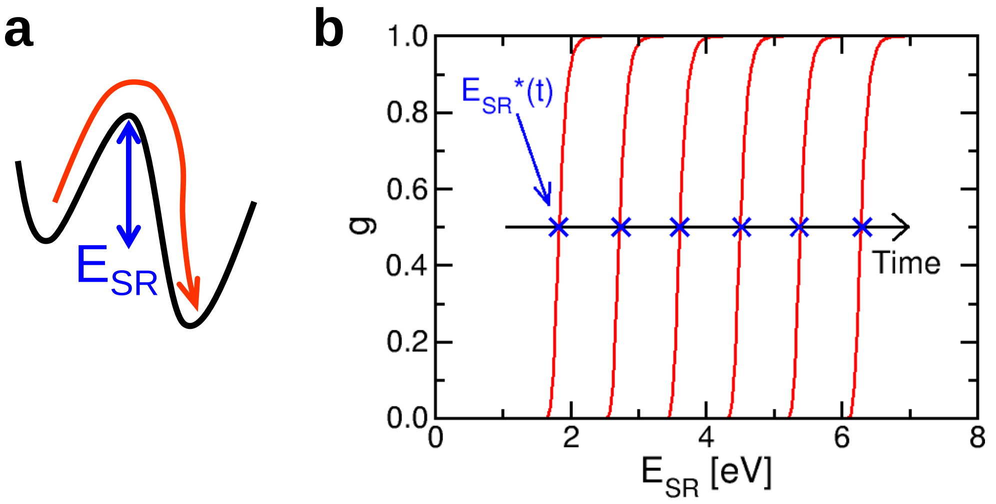

Figure S7 | Distributed energy model for resistance decay in the bipolar reset state. (a) Schematic diagram of the structural relaxation transition over a generic barrier E_{SR}, representing the single event of decay due to ion migration back to the tunneling gap. (b) Calculated generic distribution of defects $g(E_{SR})$ decaying spontaneously due to SR for increasing time [3]. The model is used to describe the stretched decrease of resistance at increasing annealing T. The annealing rate for defects is described by the formula:

$$\frac{dg}{dt} = -\frac{g}{\tau_0} e^{-\frac{E_{SR}}{kT}} \quad (1)$$

where τ_0 is a time constant and k is the Boltzmann constant. The pre-exponential constant t0 was assumed to obey to the Meyer-Neldel [4], namely:

$$\tau_0 = \tau_{00} e^{-\frac{E_{SR}}{kT_{MN}}} \quad (2)$$

Due to the Arrhenius dependence, defects at low E_C decay at relatively short time, as indicated by the time evolution of g (b). A linear relationship was assumed between the activation energy of conduction E_C and the energy E^*_{SR} marking the energy of annealed traps (b), namely:

$$E_C = \alpha E^*_{SR} \quad (3)$$

where α = 0.1 is a constant. E_C was finally used in the Arrhenius formula for the resistance of the bipolar reset state, namely:

$$R = R_0 e^{\frac{E_C}{kT}} \quad (4)$$

where R_0 is a constant. As E^*_{SR} increases due to the thermally-activated decay of the tunneling barrier, E_C and consequently R decrease. The model allows for predicting the resistance decay as a function of time and temperature and for any complicated profile of T with time, e.g., increasing T in the multiple annealing experiment in Fig. 5. This allows accurate and realistic description of resistance during device operation.

REFERENCES

1. Ielmini, D., Lacaita, A. L., Pirovano, A., Pellizzer, F. & Bez, R. Analysis of phase distribution in phase-change nonvolatile memories. *IEEE Electron Device Lett.* **25**, 507-509 (2004).
2. Ciocchini, N., Laudato, M., Leone, A., Fantini, P., Lacaita, A. L., & Ielmini, D. Universal thermoelectric characteristic in phase change memories. *IEEE – International Memory Workshop (IMW)*, Monterey, May 17-20, 2015.
3. Ielmini, D., Lavizzari, S., Sharma, D., & Lacaita, A. L. Temperature acceleration of structural relaxation in $Ge_2Sb_2Te_5$. *Appl. Phys. Lett.* **92**, 193511 (2008).
4. Ielmini, D., & Boniardi, M. Common signature of many-body thermal excitation in structural relaxation and crystallization of chalcogenide glasses. *Appl. Phys. Lett.* **94**, 091906 (2009).
